# Biases, evolutionary mismatch and the comparative analysis of human versus artificial cognition: a comment on Macmillan-Scott and Musolesi (2024)

**DOI:** 10.1098/rsos.241017

**Published:** 2025-02-26

**Authors:** Pier Luigi Sacco

**Affiliations:** ^1^ Department of Neuroscience, Imaging and Clinical Studies, University of Chieti-Pescara, Chieti 66100, Italy; ^2^ metaLAB (at) Harvard, Cambridge, MA 02138, USA

**Keywords:** biases, evolutionary mismatch, rationality, large language models

The paper by Macmillan-Scott & Musolesi [1] provides an insightful analysis of the rational reasoning capabilities of large language models (LLMs) using cognitive tasks originally designed to study human decision-making biases. Their findings reveal systematic differences between human and LLM reasoning. This raises important questions about the nature of these differences and the appropriateness of using abstract notions of rationality as benchmarks for evaluating both human and artificial cognition.

Human cognitive patterns often characterized as departures from rationality can be better understood through the lens of evolutionary mismatch—the disparity between our inherited cognitive tools and contemporary environmental demands [2,3]. Heuristics and biases that may seem irrational from a modern, abstract perspective could have been highly adaptive in the context of foraging, social coordination and survival in small-scale societies [4,5]. These cognitive tools, rather than being purely genetically specified modules, are better conceptualized as ‘cognitive gadgets’—culturally inherited thinking tools built upon genetically evolved learning capacities [6]. Over the course of human evolutionary history, our basic capacities for social learning and cultural transmission developed within specific ecological and social contexts [7], providing the foundation for more sophisticated cognitive tools that emerged through cultural evolution [8].

The apparent mismatch between these evolved cognitive strategies and contemporary demands reflects the complex interaction between biological and cultural inheritance. Our capacity for social learning evolved in ancestral environments [9,10], and the specific cognitive tools we inherited were shaped by historical contexts that differ markedly from modern conditions. As a consequence, culturally evolved cognitive-behavioural patterns, originally adaptive in past environments, may no longer be optimal for current technological and social realities [11]. Despite all this, humans feature high levels of adaptive cognitive flexibility built upon a sophisticated suite of general-purpose cognitive tools, which at least partly compensate maladaptation [12] and allow for a repurposing of preexisting cognitive adaptations to the demands of new environments [13].

This implies that we misconstrue as biases or irrational behaviour the outcome of a very complex interplay of biological, cultural and social forces at play [14,15]. Such processes do not call for ‘corrections’, but for the development of new, culturally selected socio-cognitive tools that allow us to upgrade our bio-cognitive toolkit in tailor-made ways that respond to new adaptive challenges for which we could not evolve appropriate tools at the gene–culture coevolution timescale.

These cognitive gadgets are constructed through sophisticated social learning mechanisms operating on more basic genetic predispositions [16]. Unlike genetically specified modules, they are actively built during development through cultural transmission and modification, allowing for both flexibility and historical contingency in human cognitive development [17]. The mismatch between these inherited tools and modern environments occurs because cultural evolution, while faster than genetic evolution, still lags behind rapid technological and social change [18–20].

From this perspective, the differences between human and LLM reasoning patterns reflect fundamentally different architectures of learning and development. While LLMs learn from human-generated content, they lack the embodied social learning mechanisms through which humans construct cognitive gadgets [21]. The statistical patterns they extract cannot replicate the rich process of cultural inheritance and modification that shapes human cognitive development [22]. Moreover, their training environment differs radically from the contexts that shaped our cognitive tools through cultural evolution [23].

In particular, the development of LLMs is guided by contemporary performance metrics that are unrelated to the complex cultural evolutionary processes that shaped the most fluid and flexible features of human cognition [24]. While human cognitive tools evolved through cycles of cultural transmission, modification and selection in specific social and environmental contexts, LLMs developed through optimization against abstract benchmarks [25]. This creates a fundamental distinction between culturally evolved human cognitive tools and computationally optimized LLM behaviour patterns.

Given these considerations, aligning artificial and human cognition requires understanding not just current behaviour patterns but the developmental and evolutionary processes through which human cognitive tools are constructed and transmitted [26]. This includes recognizing how the biological self-organization principles driving embodied experience [27] and the neurocognitive principles driving cultural learning [28] shape human cognitive development, and how the absence of these factors in LLMs leads to fundamentally different patterns of information processing [29,30]. Whereas LLMs may be trained to mimic evolved human behaviour in a general sense, their possible future embodiment and the behaviour they learn at a specific level are not the result of any kind of evolutionary pressure [31,32].

The findings of Macmillan-Scott & Musolesi [1] underscore the importance of considering the specific learning environments that shape the cognitive strategies of LLMs. As these models become increasingly sophisticated and integrated into decision-making processes, it is crucial to examine how their underlying architectures and training paradigms influence their reasoning patterns [33]. By taking a culturally evolutionary perspective on both human and artificial cognition, we can develop a more comprehensive understanding of the factors that shape decision-making strategies and work towards creating artificial intelligence (AI) systems that are better aligned with human goals and ecological realities [34].

In conclusion, the differences between human and LLM reasoning highlighted by Macmillan-Scott & Musolesi [1] reflect complex patterns of evolutionary mismatch operating through culturally evolved cognitive gadgets rather than genetic adaptations alone. This perspective suggests that understanding human–AI differences requires examining both the evolutionary history of our learning capacities and the cultural evolution of specific cognitive tools [35]. Such understanding becomes crucial as we develop AI systems intended to complement human cognition.

## Data Availability

This article has no additional data.
